# Thyroid Peroxidase Antibodies in Non-Interferon Treated Hepatitis C Patients in Pakistan

**DOI:** 10.1155/2015/172981

**Published:** 2015-11-03

**Authors:** Muhammad Imtiaz Shafiq, Amna Gauhar, Muhammad Akram, Shan Elahi

**Affiliations:** ^1^Institute of Biochemistry and Biotechnology, University of the Punjab, Lahore 54590, Pakistan; ^2^Institute of Chemistry, University of the Punjab, Lahore 54590, Pakistan; ^3^Department of Biochemistry, SFINHS, Shaikh Zayed Postgraduate Medical Institute, Lahore, Pakistan; ^4^Centre for Nuclear Medicine (CENUM), Mayo Hospital, Lahore 54000, Pakistan

## Abstract

*Objective*. Interferon therapy of HCV infected patients is associated with development of thyroid dysfunctions. Patients with pretreatment presence of antithyroid peroxidase (TPO-Ab) are at greater risk. This study, probably the first in Pakistan, was planned to determine TPO-Ab in sera of treatment-naive local HCV patients. *Setting*. Centre for Nuclear Medicine (CENUM), Mayo Hospital, Lahore. *Patients and Methods*. During July to December 2012, 190 patients (140 females, 50 males) newly diagnosed for HCV infection were selected for this study. Their age range was 15–55 years (mean: 35.3 ± 9.1 years). 262 age matched healthy subjects (211 females and 50 males) were recruited as control. Serum-free thyroxin (FT_4_) and thyroid stimulating hormone (TSH) were detected by radioimmunoassay techniques. Serum TPO-Ab titer was determined by ELISA method using commercial kits. *Results*. Serum FT_4_ and TSH levels in HCV patients and controls were within normal range. Between two groups there was no significant difference in mean value of FT_4_ (16.0 ± 3.0 versus 16.2 ± 3.9; *P* = 0.619) but mean TSH value was significantly lower in HCV patients (1.5 ± 0.8 versus 1.8 ± 0.9; *P* = 0.003). Among HCV patients 51 (26.8%) were TPO-Ab positive and among control subjects 18 (6.9%) were TPO-Ab positive. The difference was statistically significant (*P* < 0.001). Further analysis showed that among HCV patients 39 (27.8%) females and 12 (24.0%) males were TPO-Ab positive, respectively, and difference was not statistically significant (*P* = 0.873). Moreover, TPO-Ab positive patients were older and had significantly higher serum TSH as compared to TPO-Ab negative HCV patients. *Conclusion*. Independent of patient's gender and increasing with advancing age, about one-fourth of local untreated HCV patients are TPO-Ab positive and are at greater risk of developing thyroid disorders during and after interferon treatment.

## 1. Introduction

The world epidemiology reports of hepatitis C virus (HCV) indicate that 150–200 million people (about 3% of the world's population) are infected with HCV [1]. It is a single stranded positive RNA virus which belongs to Flaviviridae family [2]. HCV contain three structural proteins, that is, envelope 1 (E1), envelope 2 (E2), and core protein, and four nonstructural (NS) proteins, that is, nonstructural 2, 3, 4, and 5 proteins [3]. HCV causes hepatic complications that lead to chronic infection. HCV chronic infection is also related to a large number of extrahepatic manifestations including autoimmune thyroiditis [4]. Several studies suggested that hepatitis C virus infection and autoimmune thyroid diseases are linked with Th1 immune response [5]. It has been observed that hepatitis C viral nonstructural NS5 and structural protein core alone or with cytokines, that is, TNF-*α* and IFN-*γ*, provoke the upregulation of CXCL10 gene expression in the thyroid follicular cells of thyroid gland. Actually CXCL10 acts as a chemokine which attracts the lymphocytes, that is, Th1, to the site of inflammation which is caused by hepatitis C virus. The secretion of IFN-*γ* and TNF-*α* which provokes the secretion of CXCL10 by follicular cells as a result constitutes the immune cascade leading to the induction of autoimmune thyroiditis disease in genetically susceptible patients [6]. Consequently presence of CXCL10 in hepatitis C virus patient is not only related to the induction of autoimmune disease but also related to a marker of violent destructions of thyrocytes in thyroid gland. The secretion of IFN-*γ* by Th1 lymphocytes also triggers the apoptosis of thyroid epithelial cells [7].

In addition to autoimmune thyroiditis many other immunological abnormalities have also been reported in patients with chronic hepatitis C [8]. The presence of different serum autoantibodies is common in chronic HCV. These include serum nonorgan specific autoantibodies, antibodies to nuclei (ANA), smooth muscle (SMA), and liver/kidney microsomes type 1 (anti-LKM1) [8–11]. The subspecificities of these autoantibodies can be used as a diagnostic marker to distinguish between HCV and autoimmune hepatitis (AIH) [12]. It has also been reported that in some rare cases HCV can also manifest AIH features [13].

In Pakistan, local studies have reported thyroid dysfunction in up to 20% of HCV patients after IFN and ribavirin treatment [14, 15]. However, no reports are available for the evaluation of TPO-Ab in HCV patients before interferon treatment. It may be speculated that the high incidence of TD in IFN treated patients is because of preexisting TPO-Ab in HCV patients. Thus pretreatment screening for TPO-Ab is recommended for all HCV patients in whom IFN-a therapy is being planned. Presence of TPO-Ab need not be a contraindication to IFN-a therapy but its pretreatment evaluation may allow identifying the at-risk patients' true elucidation of thyroid dysfunction after IFN treatment in HCV patients.

The aims and objective of present study are to study the prevalence of TPO-Ab in HCV infected patients referred to CENUM. In addition this study highlight the difference in levels of thyroid function tests (FT_4_ and TSH) in TPO-Ab positive and negative HCV infected patients. The effects of gender, age, and serum TSH on prevalence of TPO-Ab in HCV infected patients have also been studied.

## 2. Patients and Methods

### 2.1. Patients' Selections

Records of all referred known hepatitis patients, aged 15–60 years, attending CENUM during July to December 2012 were reviewed. From them both female and male with normal FT_3_ and FT_4_ (euthyroid) were initially selected for this study. Among them such women who were already diagnosed with thyroid diseases and taking thyroid medications or had thyroid surgery were excluded. Similarly patients suffering from systematic diseases like diabetes mellitus and cardiac diseases were also excluded. We also excluded such patients whose record was not available. Serum samples of finally selected males and females were preserved for TPO-Ab determination. Previously these patients had undergone clinical assessment and determination of serum FT_4_, FT_3_, and TSH concentrations.

### 2.2. Collection of Blood Samples

An approximately 5 mL blood sample was taken from each patient. The blood sample was placed in centrifugation machine in order to separate the serum from blood for 5 minutes at low-speed centrifugation, that is, 2000 rpm at room temperature.

### 2.3. Analysis of Serum Samples for FT_4_, FT_3_, TSH, and TPO Antibodies

The serum samples which were obtained after centrifugation were stored at −20°C. Serum samples were analyzed for FT_4_, FT_3_, TSH, and TPO antibodies. FT_4_ and FT_3_ were detected by radioimmunoassay (RIA), TSH was detected by IRMA technique, and serum TPO-Ab titer in selected patients was determined by ELISA method using commercial kit of IMMCO Diagnostics, Inc., NY, USA.

RIA and IRMA batches were run with commercially manufactured control sera at different concentrations [16, 17]. Analysis of different samples, measurement of their radioactivity, and standard curve fitting were obtained by using computerized gamma counter. Assay consistency was developed by the use of commercially manufactured control sera of high, medium, and low concentrations in each run and all assays were carried out in a duplicate manner. The results of RIA and IRMA were expressed at less than 10% CV of imprecision profile. Normal ranges as standardized in our laboratory for FT_3_, FT_4_, and TSH were 2.8–5.8 pmol/L, 11–22 pmol/L, and 0.3–4.0 mIU/L, respectively. The patients with TPO-Ab titer >12.0 IU/mL were considered positive according to instructions of kit manufacturer. Microsoft Excel was used for analysis of data and chi-square test was applied for determination of the significance difference between two groups. Chi-square was applied. A *P* < 0.05 value was measured for statistical significance.

## 3. Results

A total of 231 newly diagnosed HCV patients attended CENUM during July to December 2012. Out of 231 patients, 190 (82.3%) patients had serum TSH level within normal range (0.3–4.0 mIU/L). Among them 140 were females and 50 were males. Randomly selected 262 HCV negative subjects (211 females and 50 males) were selected as controls. All of them had normal serum FT_4_ and TSH (euthyroid).

The demographic and clinical characteristics of HCV patients and control subjects are shown in Table 1.

As compared to healthy controls, HCV infected patients were comparatively older and had low mean serum TSH. Serum TPO-Ab was determined in sera of both case and control subjects. The incidence of TPO-Ab positivity was 51 (26.8%) and 18 (6.9%) in HCV patients and control healthy subjects, respectively. The difference was statistically significant (*P* < 0.001).

Table 2 shows the comparison of demographic and clinical characteristics of TPO-Ab positive subjects between both groups of HCV patients and healthy controls. Similar to the finding reported in Table 1, HCV infected TPO-Ab positive patients were comparatively older and had low mean serum TSH as compared to TPO-Ab positive control subjects. Among HCV patients 39 (27.8%) females and 12 (24.0%) males were TPO-Ab positive, respectively. The difference was not statistically significant (*P* = 0.873). Similarly among control subjects difference between female and male was not statistically significant (*P* = 0.969). To elucidate the effect of patients age and serum TSH on incidence of TPO-Ab, a comparison of TPO-Ab positive and TPO-Ab negative HCV patients is shown in Table 3. TPO-Ab positive patients were significantly older and had higher serum TSH as compared to TPO-Ab negative patients. In summary, incidence of TPO-Ab positivity among HCV patients is 26.8% which is significantly higher as compared to healthy adult population. TPO-Ab positivity is independent of HCV patient gender but is higher in patients with advanced age and raised serum TSH.

## 4. Discussion

The aim of this study was to know the prevalence of TPO-Ab in euthyroid HCV patients referred to CENUM, Mayo Hospital, for thyroid function testing before interferon treatment. In a selected data set of HCV patients the incidence of TPO-Ab was detected in 51 (26.8%). Other studies have reported TPO-Ab prevalence in 2% to 30% of non-interferon treated HCV patients [18–21]. Present study results are in accordance to Yang et al. (2011) who reported TPO-Ab prevalence in 30.8% of HCV patients before INF-a treatment [20]. The prevalence of TPO-Ab in 21% of patients has also been reported in HCV infection [21]. The incidence of TPO-Ab positivity was significantly higher (*P* < 0.0001) as compared to healthy euthyroid adult population that had incidence of 6.9%. This is in accordance with recent studies [21, 22] but some older studies do not support this finding [23, 24]. The difference may be due to improvement in laboratory diagnosis of thyroid autoantibodies.

Different studies have proposed several mechanisms for induction of autoimmune thyroiditis by HCV infection like molecular mimicry between viral antigens and self-antigens, activation of autoreactive T cells (bystander mechanism) by induction of local inflammation, induction of aberrant expression of MHC class II molecules on thyroid cells, viral induction of changes in self-antigen expression of cryptic epitopes, and induction of heat-shock proteins in the thyroid [4, 25, 26]. On the other hand, the HCV inducing the thyroid autoimmunity in predisposed individual are still mysterious. Among abovementioned mechanisms, two hypotheses, that is, molecular mimicry and bystander activation, have strongest indication of development of thyroid autoimmunity in hepatitis C virus patients. The molecular mimicry hypothesis suggested that sequence of nucleotides similar between self-proteins and viral proteins can cause a cross over immune reaction to self-antigens which are imitated by “infectious agents” proteins [26, 27]. Though HCV could not transmit a virus to thyrocytes (thyroid gland cells), coat of virus which is basically a protein also influenced the other body parts because it has a significant physiological effects. For instance, two HCV structural proteins, that is, E1 and E2, have a capability to bind with certain body surface molecules, that is, CD81, which help in viral entry [27]. According to bystander activation hypothesis, local inflammation is induced by viral infection; as a result, stimulation of autoreactive T lymphocytes occurs which were repressed by Treg cells (regulatory T cells) through peripheral tolerance mechanisms [27]. Recent facts support the bystander activation process as the crucial method by which hepatitis C virus induces autoimmune thyroiditis. Consequently, it is proved that the relation among thyroid autoimmunity and hepatitis C virus infection is caused by HCV infection of the thyroid as a result of proinflammatory mediators release, such as IL-8, and development of thyroid autoimmunity through bystander activation process [4].

Thyroid autoimmunity is enhanced in female as compared to male subjects [28]. The similar trend is reported in HCV infected patients by different studies [20, 29]. However, according to recent study results between HCV patients 39 (27.8%) and 12 (24.0%) females and males were TPO-Ab positive, respectively. The difference was not statistically significant (*P* = 0.873). Similarly among control subjects difference between female and male was not statistically significant (*P* = 0.969). Thus TPO-Ab positivity was independent of patient's gender. This is in accordance with some studies [19, 30] but most studies have reported more incidence of TPO-Ab in female as compared to male HCV patients [18, 22, 31]. Similarly, Hass et al. (2009) detected significantly more abnormal pretreatment TSH levels (TSH > 3.0 mU/L or <0.4 mU/L) in female patients and Testa et al. (2006) reported almost double incidence of hypothyroidism in female as compared to male patients [32, 33]. The apparent reason for disagreement between our study and other studies may be the difference in patients' selection method. TPO-Abs were determined in referred HCV patients while other studies had recruited HCV positive patients without considering signs and symptoms of thyroid dysfunction. Higher incidence of TPO-Ab positivity in patients with advanced age is observed in both HCV patients and control and the same trend was also found in TPO-Ab positive HCV patients when compared with TPO-Ab positive controls. This is in accordance with another study [22]. A similar study like ours also noted a higher incidence of TPO-Ab positivity in patients presenting with raised serum TSH.

## 5. Conclusion

Thyroid gland dysfunction has been reported to occur with variable frequency during Interferon Alfa (IFN-*α*) therapy in TPO-Ab positive patients with the hepatitis C virus (HCV) [34]. The results of such studies conducted locally should be interpreted in the light of our results where 25% of HCV infected patients have TPO-Ab before this treatment and such patients are at increased risk of developing thyroid dysfunction after Interferon Alfa (IFN-*α*) therapy.

## Figures and Tables

**Table 1:** Characteristics of HCV patients and control subjects.

	Cases	Control	*P* value^*∗*^
Number (*n*)	190	200	—
Mean age (year)	35.0 ± 9.7	33.3 ± 9.9	0.083
Age range (year)	5–55	18–60	—
Number of female patients	140 (73.7%)	149 (74.5%)	0.200
Number of male patients	50 (26.3%)	51 (25.5%)	0.192
Mean FT_4_	16.0 ± 3.0	16.0 ± 2.3	0.981
Mean TSH	1.5 ± 0.8	1.7 ± 0.9	0.091
TPO-Ab positive	51 (26.8%)	17 (8.5%)	0.0001

^*∗*^ *P* value = it explains the probability of incidence of given event.

**Table 2:** Effect of HCV patient factors on TPO-Ab positivity.

	Group	TPO-Ab+	*P* value
Gender	Female (*n* = 140)	39 (27.8%)	0.873
	Male (*n* = 50)	12 (24.0%)	

Age	≤35 Y (*n* = 102)	24 (23.5%)	0.536
	>35 Y (*n* = 88)	27 (30.7%)	

TSH	<2.5 (*n* = 161)	39 (24.2%)	0.161
	≥2.5 (*n* = 29)	12 (41.4%)	

**Table 3:** Comparison of TPO-Ab positive and TPO-Ab negative HCV patient.

Characteristics	Group		*P* value^*∗*^
	TPO-Ab positive	TPO-Ab negative	
Number (*n*)	51	139	
Mean age (year)	37.8 ± 9.2	34.3 ± 9.6	0.027
Mean FT_4_ (pmol/L)	15.6 ± 2.8	16.1 ± 3.0	0.348
Mean TSH	1.8 ± 0.9	1.4 ± 0.7	0.012

^*∗*^ *P* value = it explains the probability of incidence of given event.
